# Patterns and associated factors of shisha usage among the undergraduate students of Jouf University, Saudi Arabia: A cross-sectional study

**DOI:** 10.18332/tid/186185

**Published:** 2024-05-28

**Authors:** Rakhi Issrani, Danah Sultan R. Alruwaili, Rola H. G. Alruwaili, Robina Tasleem, Abdulmajeed Almuaddi, Khalid M. Abdelaziz, Shahad M. M. Alruwaili, Naif Sultan R. Alruwaili, Alzarea K. Bader, Zafar A. Khan, Namdeo Prabhu

**Affiliations:** 1 College of Dentistry, Jouf University, Al-Jawf, Kingdom of Saudi Arabia; 2 College of Dentistry, King Khalid University, Abha, Kingdom of Saudi Arabia; 3 Dentistry Academic Department, Vision College, Riyadh, Kingdom of Saudi Arabia; 4 Department of Oral and Maxillofacial Surgery, Frontier Medical and Dental College, Abbottabad, Pakistan; 5 Department of Research Analytics, Saveetha Dental College and Hospitals, Saveetha Institute of Medical and Technical Sciences, Saveetha University, Chennai, India

**Keywords:** shisha, Saudi Arabia, smoking students

## Abstract

**INTRODUCTION:**

Shisha tobacco use is gaining popularity around the world, especially among young people and college students. Shisha users are exposed to many of the same harmful substances as cigarette smokers but at much higher levels, which could have more serious adverse health impacts. The aims of this study were to: 1) determine the patterns of shisha smoking among university students in the Northern Province of Saudi Arabia; 2) identify the reasons for using shisha tobacco; and 3) ascertain whether usage of shisha smoking differs by their sociodemographic characteristics.

**METHODS:**

A cross-sectional survey was conducted among students of seven different colleges at a public university between October and December 2022. A modified version of the Global Youth Tobacco Survey questionnaire was used to collect information specifically on shisha smoking. After adjusting for confounding variables, a logistic regression analysis was used to determine the related factors.

**RESULTS:**

A total of 418 participants were included in the study. Of the studied population, shisha smoking was seen in 73.7% (n=308). Out of 308 shisha smokers, 208 (67.5%) had their first session of shisha smoking within the past two years; 34.4% of participants had used it in the past 30 days (current users), of which 27.4% had smoked shisha from 1 to 7 days during the past 30 days. The majority of respondents (63.6%) reported having friends that smoke. The most popular flavor among respondents (24.6%) was apple. For quitting attempts, 46.4% reported a 24-hour quit attempt in the previous year. Stress (23.0%) was reported as the main reason for beginning shisha use. Shisha smoking was significantly associated with age (Ref. 18–20 years; 24–26 years, AOR=0.08; 95% CI: 0.02–0.33, p<0.001; ≥27 years, AOR=0.12; 95% CI: 0.02–0.62, p=0.01), living status (Ref. alone; with family, AOR=0.23; 95% CI: 0.11–0.47, p<0.001; with friends, AOR=0.36; 95% CI: 0.18–0.76, p<0.001), with parents having higher education level (Ref. primary school and lower; Bachelor’s, AOR=0.33; 95% CI: 0.14–0.76, p<0.001; diploma, AOR=0.33; 95% CI: 0.15–0.73, p<0.001; PhD, AOR=5.15; 95% CI: 1.00–9.65, p=0.05).

**CONCLUSIONS:**

Shisha use was more frequent among Saudi Arabian college students, especially those who were aged 18–20 years, living alone, and having highly educated parents.

## INTRODUCTION

Shisha is a tobacco pipe with a long, flexible tube that draws the smoke through water held in a bowl. Other names include waterpipe, narghile, chica, hubble bubble, and hookah^1^. A recent systematic review on the prevalence of shisha smoking reported prevalence rates of 15–28% in the East Mediterranean region, 33% in the South Asia region, 10% in the Americas, and 8% in Europe^2^. Shisha smoking has become more common, particularly among college and high school students^3^. This rise in popularity is partly attributable to the myth that shisha filters the smoke, making it less harmful than other tobacco smoking methods^4^. In fact, shisha smoke includes nicotine, which leads to addiction; high levels of carbon monoxide which contribute to cardiovascular disease; and harmful aldehyde compounds which can cause lung inflammation^5^. As a result, shisha smoking raises the risk of developing cancer, lung disease, cardiovascular disease, dependence, and other issues^3^. Also, it is worth noting smoking shisha exposes a person to nicotine nine times longer than smoking a cigarette (45 minutes vs 5 minutes) and nearly twice as high levels (1.7 times) as smoking a cigarette^6^. Additionally, shisha smokers utilize tobacco that has been flavor-infused, which makes smoking shisha far more alluring^6^. Moreover, shisha smoking also carries the cross-infection risk by sharing a shisha mouthpiece^7^.

In Saudi Arabia, more than 20000 children (aged 10–14 years) and 3.35 million people (aged ≥15 years) continue to use tobacco every day, and the country experiences more than 7000 tobacco-related deaths each year, according to the Tobacco Atlas^7^. Shisha tobacco usage is expanding globally, especially in Saudi Arabia and the Eastern Mediterranean, even though cigarette smoking has decreased as a result of significant public health campaigns^8^. Compared to its neighbors, Saudi Arabia records more instances of shisha use^9,10^. In 2010, the most recent Saudi Arabia global youth tobacco survey of students aged 13–15 years found that 9.5% of students in intermediate grades 1 through 3 currently smoked shisha^7^.

There is a severe public health concern in Saudi Arabia regarding the absence of effective interventions that specifically target young people who smoke shisha^7^. The World Health Organization Study Group on Tobacco Product Regulation states that more research is required on all aspects of shisha smoking, including the types and patterns of shisha consumption in all regions and cultures^11^. Traditional cigarettes have typically been the focus of cigarette smoking research and control initiatives, but shisha smoking has received less attention. The use of shisha among the general public has also been the subject of some studies; however, there has not been much research on its use among college students.

To fill these gaps in the literature, the current study aimed to determine the patterns and factors associated with shisha tobacco smoking among university students in Saudi Arabia, in addition to identifying the reasons for using shisha tobacco and ascertaining whether usage of shisha smoking differs by sociodemographic characteristics.

## METHODS

### Study design

A cross-sectional study was used with data collected between October and December 2022. Ethical approval (no. 10-10-43) was obtained from the Local Committee of Bioethics, Jouf University, and all the procedures in this study were in compliance with the Helsinki Declaration.

### Sample population and characteristics

We targeted a sample of 500 students from different colleges at Jouf University, Saudi Arabia, which is one of the leading public universities in the northern region. Participants who met the inclusion criteria for participation were chosen by non-probability convenience sampling. Only Saudi nationals who were aged ≥18 years, male or female, and enrolled at one of the colleges of Jouf University were included. Students unwilling to participate were excluded from the study.

### Survey tool

We used a specific structured questionnaire modified from the Global Youth Tobacco Survey questionnaire, an international standardized questionnaire developed by the World Health Organization^12^. In total, there were seventeen questions. The questionnaire was divided into the following four sections:

1. Section I collected information about participant’s characteristics such as age, gender, student specialization, academic year, marital status, living status, and parental education level (a proxy for socioeconomic status).
2. Section II was intended to assess the prevalence of smoking shisha. The participants were asked if they had ever tried or experimented with smoking shisha, even one or two puffs (the options were ‘yes’ or ‘no’).
3. Section III focused on patterns of shisha smoking (Questions 9–16). The question of whether the participants had first tried shisha during the previous two years or more than two years was put to the participants. When asked how frequently they smoked shisha, respondents had three options: ‘used but not in the past 12 months’, ‘used but not in the past 30 days’, or ‘used in the past 30 days’. Students who reported consuming shisha within the preceding 30 days were classified as current users. The frequency of smoking of present users was inquired (‘On how many days in the last 30 days did you smoke shisha?’). To measure peer influence, students were asked: ‘Do you usually smoke alone or primarily when you are with others?’. The students who frequently smoked with their friends were asked how many hours per day they smoked ( >2 or ≤2). Additionally, the shisha smokers were requested their favorite flavors.

Smokers were asked how often they had attempted to quit smoking shisha in the year prior and had been successful for at least 24 hours. The validated Stages of Change scale was employed to measure the intention to stop smoking within the next 30 days or six months. Precontemplation (not planning to quit in the next six months), contemplation (planning to quit in the next six months but not the next 30 days), and preparation (planning to quit in the next 30 days and have made a successful 24-hour quit attempt in the previous year) were used to categorize smokers into these three stages^13,14^.

1. Section IV was related to the reasons for starting shisha smoking.

### Mailing and response rate

In October 2022, 500 students were mailed surveys in Google Survey Forms. Within three weeks, a questionnaire and a reminder email were both delivered. A disclaimer regarding informed consent was presented at the start of the survey form, and participation was voluntary. The replies were anonymous.

### Reliability of questionnaire

The questionnaire was piloted on 15 fourth-year dental students (who were not included in the main study) to ensure its clarity, practicability, and ease of completion. Based on the findings of this pilot study, the questionnaire’s reliability was assessed using Cronbach’s alpha, which was 0.82.

### Statistical analysis

Data were analyzed using SPSS 20.0 (IBM Corp., Armonk, NY, United States). Quantitative data are presented as frequencies and percentages. A multivariate regression model was applied to identify the independent determinants of shisha smoking (ever smokers of shisha vs non-smokers of shisha). In the logistic regression model, the above-mentioned binary outcome variables were compared with the potential variable, as included in the data collection form. Results are presented as adjusted odds ratio (AOR) and their 95% confidence interval (CI). A p<0.05 was set as a statistically significant value^15^.

## RESULTS

### Baseline characteristics of participants

The response rate was 83.6% (N=418). Table 1 shows the participants’ sociodemographic details. Among the 418 respondents, 54.5% were female, 49.3% were aged 21–23 years, 29.9% were dental students, 30.9% were in their first year of study, 95.2% were unmarried, and 38.0% lived with family. The majority of the participants’ parents were diploma holders (24.4%). The majority (73.7%) of the participants were ever smokers of shisha, while 26.3% were non-smokers of shisha.

**Table 1:** Sociodemographic characteristics of the students of seven different colleges at a public university from a survey regarding shisha smoking usage, conducted between October and December 2022 in Saudi Arabia (N=418)

*Characteristics*	*n*	*%*
**Age** (years)		
18–20	134	32.1
21–23	206	49.3
24–26	62	14.8
≥27	16	3.8
**Gender**		
Male	190	45.5
Female	228	54.5
**College**		
Dental	125	29.9
Medical	114	27.3
Engineering	41	9.8
Law	30	7.2
Nursing	37	8.9
Business management	51	12.2
Pharmacy	20	4.8
**Academic year**		
First	129	30.9
Second	58	13.9
Third	70	16.7
Fourth	100	23.9
Fifth	36	8.6
Intern	25	6.0
**Marital status**		
Married	20	4.8
Unmarried	398	95.2
**Living status**		
Alone	136	32.5
With family	159	38.0
With friends	123	29.4
**Parental education level**		
Primary school and lower	93	22.2
Secondary school	92	22.0
Bachelor’s	86	20.6
Diploma	102	24.4
Master’s	28	6.7
PhD	17	4.1

### Patterns of shisha smoking

Out of 308 shisha smokers, 208 (67.5%) had their first session of shisha smoking within the past two years. In terms of frequency of use, 34.4% of participants had used it in the past 30 days (current users), of which 27.4% had smoked shisha from 1 to 7 days during the past 30 days. The majority of the respondents had friends who had the habit of smoking (63.6%). Shisha smoking was more common among smokers who spent more time socializing with friends, especially those who spent >2 hours per day with their smoking friends (43.5%). Apple was the most favored flavor of the respondents (24.6%). For quitting attempts, 46.4% reported a 24-hour quit attempt in the previous year, but the majority of shisha smokers (53.6%) were not planning to quit smoking shisha. Of all the participants who had at least one 24-hour attempt to quit shisha smoking, 18.5% were in the pre-contemplation stage, i.e. no plan to quit in the next six months, whereas 16.2% belonged to the contemplation stage, i.e. plan to quit in within six months and 11.7% planned to quit within 30 days. Table 2 demonstrates the patterns of shisha smoking among the participants.

**Table 2:** Patterns of shisha smoking amongst students of seven different colleges at a public university in Saudi Arabia, from a survey conducted between October and December 2022 (N=308)

*Patterns*	*Shisha smokers*	
	*n*	*%*
**Onset of shisha smoking**		
First session within the past 2 years	208	67.5
First session >2 years ago	100	32.5
**Frequency of shisha use**		
Used but not in the past 12 months	117	38.0
Used but not in the past 30 days	85	27.6
Used in the past 30 days (current users)	106	34.4
**During the past 30 days, how many days did you smoke shisha?** (N=106)		
0	28	26.4
1–7	29	27.4
8–14	18	17.0
15–29	16	15.1
30	15	14.1
**Peer influence**		
With smoking friends	196	63.6
Without smoking friends	112	36.4
**Socializing with smoking friends**		
>2 hours per day	134	43.5
≤2 hours per day	62	20.1
**Flavors of shisha**		
Apple	76	24.6
Plum	48	15.6
Coconut	45	14.6
Mango	41	13.3
Mint	36	11.7
Strawberry	23	7.5
Cola	39	12.7
**Quitting attempt**		
No attempt	165	53.6
At least one 24-h quit attempt in past year	143	46.4
**Stage of quitting**		
No attempt	165	53.6
Precontemplation (no plan to quit in the next 6 months)	57	18.5
Contemplation (plan to quit within 6 months)	50	16.2
Preparation (plan to quit within 30 days)	36	11.7

According to Table 3, which lists the reasons students start smoking shisha, stress (23.0%) was the most common, followed by peer pressure (16.5%), curiosity (14.4%), boredom (12.0%), and pleasure-seeking (7.9%).

**Table 3:** Reasons reported for starting shisha smoking among students of seven different colleges at a public university in Saudi Arabia, from a survey conducted between October and December 2022 (N=308)

*Factors*	*Shisha smokers*	
	*n*	*%*
Stress	96	23.0
Boredom	50	12.0
Peer pressure	69	16.5
Pleasure-seeking	33	7.9
Curiosity	60	14.4

### Factors associated with shisha smoking behavior

The binary logistic regression analysis revealed that the shisha smoking was significantly associated with age (Ref. 18–20 years; 24–26 years, AOR=0.08; 95% CI: 0.02–0.33, p<0.001; ≥27 years, AOR=0.12; 95% CI: 0.02–0.62, p=0.01), living status (Ref. alone; with family, AOR=0.23; 95% CI: 0.11–0.47, p<0.001; with friends, AOR=0.36; 95% CI: 0.18–0.76, p<0.001), with parents having higher education level (Ref. primary school and lower; Bachelor’s, AOR=0.33; 95% CI: 0.14–0.76, p<0.001; diploma, AOR=0.33; 95% CI: 0.15–0.73, p<0.001; PhD, AOR=5.15; 95% CI: 1.00–9.65, p=0.05). Table 4 shows the factors associated with shisha smoking behavior.

**Table 4:** Factors associated with shisha smoking behavior among students of seven different colleges at a public university in Saudi Arabia, from a survey conducted between October and December 2022 (N=418)

*Variables*	*Non-smokers of shisha (N=110)*	*Shisha smokers (N=308)*						
	*n (%)*	*n (%)*	*OR*	*95% CI*	*p*	*AOR*	*95% CI*	*p*
**Age (years)**								
18–20 ®	18 (4.3)	116 (27.8)	1			1		
21–23	43 (10.3)	163 (39.0)	0.59	0.32–1.07	0.08	0.87	0.29-2.67	0.81
24–26	39 (9.3)	23 (5.5)	0.09	0.05–0.19	0.01*	0.08	0.02–0.33	<0.001*
≥27	10 (2.4)	6 (1.4)	0.09	0.03–0.29	0.01*	0.12	0.02–0.62	0.01*
**Gender**								
Male ®	45 (10.8)	145 (34.7)	1			1		
Female	65 (15.6)	163 (39.0)	0.78	0.50–1.21	0.27	0.64	0.37–1.12	0.12
**College**								
Dental ®	28 (6.7)	97 (23.2)	1			1		
Medical	38 (9.1)	76 (18.2)	0.58	0.33–1.02	0.06	0.65	0.31–1.35	0.25
Engineering	12 (2.9)	29 (6.9)	0.69	0.32–1.54	0.37	0.50	0.19–1.29	0.15
Law	6 (1.4)	24 (5.7)	1.16	0.43–3.10	0.78	0.73	0.24–2.22	0.58
Nursing	10 (2.4)	27 (6.5)	0.78	0.34–1.80	0.56	0.71	0.26–1.93	0.50
Business management	9 (2.2)	42 (10.0)	1.35	0.59–3.10	0.48	1.11	0.42–2.92	0.84
Pharmacy	7 (1.7)	13 (3.1)	0.53	0.19–1.47	0.22	0.74	0.22–2.49	0.63
**Academic year**								
First ®	18 (4.3)	111 (26.6)	1			1		
Second	8 (1.9)	50 (12.0)	1.01	0.41–2.49	0.98	1.02	0.31–3.34	0.97
Third	22 (5.3)	48 (11.5)	0.35	0.17–0.72	0.01*	0.41	0.12–1.38	0.15
Fourth	29 (6.9)	71 (17.0)	0.39	0.21–0.77	0.01*	0.62	0.18–2.10	0.44
Fifth	14 (3.3)	22 (5.3)	0.26	0.11–0.59	0.01*	0.63	0.15–2.59	0.52
Intern	19 (4.5)	6 (1.4)	0.05	0.0–0.15	0.01*	0.33	0.06–1.69	0.18
**Marital status**								
Married ®	10 (2.4)	10 (2.4)	1			1		
Unmarried	100 (23.9)	298 (71.3)	2.98	1.21–4.97	0.02*	1.02	0.27–3.83	0.98
**Living status**								
Alone ®	19 (4.5)	117 (28.0)	1			1		
With family	52 (12.4)	107 (25.6)	0.33	0.19–0.60	0.0*	0.23	0.11–0.47	<0.001*
With friends	39 (9.3)	84 (20.1)	0.35	0.19–0.65	0.01*	0.36	0.18–0.76	<0.001*
**Parental education level**								
Primary school and lower ®	16 (3.8)	77 (18.4)	1			1		
Secondary school	18 (4.3)	74 (17.7)	0.85	0.41–1.80	0.68	0.88	0.36–2.16	0.79
Bachelor’s	29 (6.9)	57 (13.6)	0.41	0.20–0.82	0.01*	0.33	0.14–0.76	<0.001*
Diploma	37 (8.9)	65 (15.6)	0.37	0.19–0.72	0.01*	0.33	0.15–0.73	<0.001*
Master’s	7 (1.7)	21 (5.0)	0.62	0.23–1.71	0.36	1.59	0.47–5.41	0.45
PhD	3 (2.7)	14 (4.5)	0.98	0.25–3.78	0.97	5.15	1.00–9.65	0.05*

AOR: adjusted odds ratio. ® Reference categories.

## DISCUSSION

The objective of this study was to assess the patterns and factors associated with shisha smoking among university students in Saudi Arabia. The majority of the participants were shisha smokers, and most of them had their first session of shisha smoking within the past two years. Furthermore, the majority of respondents said they had no plans to stop smoking shisha. Stress and peer pressure were the main drivers of beginning shisha use. Shisha use was significantly associated with age, living situation, and having educated parents.

Even though studies have generally indicated a significant range in the usage of shisha, epidemiological data on shisha smoking in Saudi Arabia have shown alarming evidence of high shisha usage among teenagers and college students^16^. For instance, research among university students in the Saudi Arabian city of Buraidah revealed that 46.6% of them smoked shisha^1^. Similar investigations were carried out by Taha et al.^17^, Al-Turki^18^, Albangy et al. ^19^, Alzohairy^20^ and Al Moamaryet al.^21^, and they revealed that the usage rates of smoking shisha were 12.6%, 13.0%, 22.4%, 40.0%, and 65.9%, respectively. Shisha smoking usage was found to be greater in the current study (73.7%) when compared to these investigations. The increased usage in this study may be because the previous studies reported the usage of current shisha smokers, while this study is reporting the usage of ever smokers of shisha (past and current). Shisha smoking is becoming more and more popular despite the Saudi government’s ongoing efforts to ban the sale of tobacco products in different regions of the kingdom.

Shisha smoking accounted for 53.9% of current tobacco users in Saudi Arabia, according to Amin et al.^22^. Similar rates were reported among college^23^ and medical and dental students^24-26^. In this study, the onset of shisha smoking was twice as frequent among recent-onset smokers (67.5%) compared to the individuals who had smoked for more than two years (32.5%). Regarding the frequency of shisha usage, 34.4% of participants had used it in the past 30 days (current users), of which 27.4% had smoked shisha from 1 to 7 days during the past 30 days. This finding can be attributed to the fact that some people use private locations where they can assemble for talking and smoking, such as Estrahas, in addition to an increasing number of shisha lounges and cafes. Some Estrahas use it specifically to play cards, play video games, and smoke shisha with friends. This calls into question whether efforts to reduce shisha smoking, a popular social activity, should be given higher priority in public health programs.

In their study, Moran et al.^13^ concentrated on the concept of ‘social smoking’, which refers to the practice of people smoking more when they are with others than when they are alone. They discovered that this was one of the factors contributing to the smoking epidemic among college students. Similar findings were made by Li et al.^27^ who found that students who had smoking friends were more likely to smoke than other students. The highest risk factors for tobacco use among dental students at King Saud University in Riyadh were having smoker acquaintances and being male^28^. According to research by Amin et al.^22^, smoking shisha is largely influenced by factors including gender, age, and having relatives and friends as smokers. This is consistent with the findings of our study, as 63.6% of shisha smokers did so with friends. Additionally, we discovered that students who spent more than two hours a day socializing with their smoking friends smoked more cigarettes (43.5%) than those who spent less time with their smoking friends (20.1%), probably because they had more opportunities to smoke in public.

In a recent study conducted by Salih et al.^29^ among university students in Jazan, Saudi Arabia, 50% of the participants said that smoking shisha with flavors, such as apple, makes it less dangerous than with flavorless. According to the review article of Qasim et al.^30^, flavored tobacco, which gives the user a pleasant taste and aroma, also plays a vital role as a ‘motivator’ for consuming shisha. In our study, the majority of respondents’ preferred flavor was apple (24.6%). As a result, the development of flavored tobacco, the popularity of coffee shops, and the absence of laws governing shisha smoking could all be factors in the expansion of the practice across the country.

In a survey by Kabbash and Saied^31^, 34.1% of shisha users were considering quitting. In the study by Hussain et al.^32^ in the Saudi Arabian region of Arar, 57.8% of the smoker students expressed their intention to stop smoking. Additionally, 66.7% of the investigated smoker medical students stated having the intention to give up smoking, according to Abo el-Fetoh et al.^33^. These numbers are lower than those of Stramari et al.^34^, who found that 87.2% of participants who smoke wished to stop doing so. In the current study, 46.4% of participants reported making a 24-hour attempt to quit smoking, although the majority of shisha users (53.6%) did not want to discontinue. Of all the participants who made at least one 24-hour attempt to stop using shisha, 18.5% were in the pre-contemplation stage, meaning they had no plans to stop in the next six months; 16.2% were in the contemplation stage; and 11.7% had plans to stop in the next 30 days.

College students usually choose to follow their peers’ smoking habits to socially fit in. They now associate smoking with a social stigma and a connection to their peers. In addition to having to deal with intense competition and having stress while in college, as well as the pressure from their families to do well in their studies, college students may become quickly addicted to smoking, besides their curiosity about new things and new experiences^15,27^. Peer pressure, according to Stramari et al.^34^ and Kabbash and Saied^31^, was the driving force behind smoking for 42.3% and 63.1% of smokers, respectively. In contrast, the majority of students in a study by Awan et al.^2^ said that their habit of smoking shisha was primarily driven by curiosity and pleasure-seeking. In a comprehensive study of the medical literature that looked at motives, beliefs, and attitudes towards the practice, the primary motivations for smoking shisha were identified as socializing, relaxation, enjoyment, and entertainment. Peer pressure, fashion, and curiosity were additional driving forces for college and high school students. Still, people from the Middle East and people of Middle Eastern descent living in Western countries reported additional driving forces connected to the expression of their cultural identities^9^. In the current study, the reasons for shisha smoking were mainly stress (23.0%), followed by peer pressure (16.5%), curiosity (14.4%), boredom (12.0%), and pleasure-seeking (7.9%). This could be because most of the participants in our study were pursuing medical and dental courses that are 6 to 7 years long and are very stressful in terms of academic achievement and clinical work.

In the present study, binary logistic regression was used to assess the factors associated with shisha smoking while considering a wide range of sociodemographic characteristics. It is interesting to note that in our study, participants with well-educated parents (a proxy for socioeconomic status) were more likely to smoke shisha. This could be due to the fact that it might be easily affordable by people with high socioeconomic status because the Saudi government has recently imposed a 100% excise tax on tobacco products^35^.

### Limitations

Limitations of the present study include the cross-sectional design of the study, and therefore, determining causality may not be possible. However, given that there is a shortage of literature in this area, our findings may help direct future research by assisting in the formulation of hypotheses and by providing crucial information to target susceptible populations. Another limitation is that the participants were chosen using a convenience sample and hence may not be generalizable to other populations. However, the sample was chosen from several colleges, and the large sample size ensured that students from diverse socioeconomic backgrounds were well represented^15^. Additionally, because only one type of tobacco smoking was studied and data was only gathered from one center in one nation, there is limited scope for extrapolating the findings to other nations.

## CONCLUSIONS

Shisha smoking was found to be associated with age, living alone, and having highly educated parents. Multidisciplinary health education initiatives for raising awareness among various age groups to prevent young people from smoking any kind of tobacco, including shisha, is recommended. Health policymakers must also ensure that the laws are upheld by conducting policy analyses, implementing them, and enhancing regulatory controls.

## Data Availability

The data supporting this research are available from the authors on reasonable request.
